# Theta Oscillations and Source Connectivity During Complex Audiovisual Object Encoding in Working Memory

**DOI:** 10.3389/fnhum.2021.614950

**Published:** 2021-03-08

**Authors:** Yuanjun Xie, Yanyan Li, Haidan Duan, Xiliang Xu, Wenmo Zhang, Peng Fang

**Affiliations:** ^1^School of Education, Xin Yang College, Xinyang, China; ^2^Department of Radiology, Xijing Hospital, Fourth Military Medical University, Xi'an, China; ^3^Department of Fundamental, Army Logistical University, Chongqing, China; ^4^Department of Social Medicine and Health and Management, College of Military Preventive Medicine, Army Medical University, Chongqing, China; ^5^Department of Military Medical Psychology, Fourth Military Medical University, Xi'an, China

**Keywords:** working memory, audiovisual object, encoding, theta, functional connectivity, EEG

## Abstract

Working memory is a limited capacity memory system that involves the short-term storage and processing of information. Neuroscientific studies of working memory have mostly focused on the essential roles of neural oscillations during item encoding from single sensory modalities (e.g., visual and auditory). However, the characteristics of neural oscillations during multisensory encoding in working memory are rarely studied. Our study investigated the oscillation characteristics of neural signals in scalp electrodes and mapped functional brain connectivity while participants encoded complex audiovisual objects in a working memory task. Experimental results showed that theta oscillations (4–8 Hz) were prominent and topographically distributed across multiple cortical regions, including prefrontal (e.g., superior frontal gyrus), parietal (e.g., precuneus), temporal (e.g., inferior temporal gyrus), and occipital (e.g., cuneus) cortices. Furthermore, neural connectivity at the theta oscillation frequency was significant in these cortical regions during audiovisual object encoding compared with single modality object encoding. These results suggest that local oscillations and interregional connectivity *via* theta activity play an important role during audiovisual object encoding and may contribute to the formation of working memory traces from multisensory items.

## Introduction

Working memory enables the maintenance and manipulation of information for short periods of time after a physical stimulus is no longer available (Baddeley, 1992) and is essential to many high-level cognitive functions (Christophel et al., 2017; Funahashi, 2017). In cognitive processing, a stimulus can be encoded into working memory, stored through some form of maintenance of the memory trace, and ultimately retrieved to perform a goal-oriented task. Neurocognitive models of working memory support the existence of encoding, maintenance, and retrieval stages as being necessary for successful working memory processes (D'esposito, 2007; Baddeley et al., 2011). Many functional magnetic resonance imaging (fMRI) and positron-emission tomography (PET) studies have investigated the neural mechanisms that underlie working memory at the systems level. These studies have highlighted multiple functionally relevant brain regions including the prefrontal cortex and posterior cortical areas (e.g., parietal, temporal, and occipital) as being critical to working memory functioning (Constantinidis and Procyk, 2004; Rottschy et al., 2012; D'esposito and Postle, 2015). Encoding is critical to working memory processes (Jensen and Lisman, 2005; Cohen et al., 2014) because precise encoding significantly affects the formation of memory traces and subsequent memory processes (e.g., maintenance and retrieval) after the encoded stimuli disappear, whereas encoding deficits in working memory impair behavioral performance and involve abnormally functioning brain networks (Spellman et al., 2015; Wiesman et al., 2016).

A fundamental question in working memory research is how neural populations encode and represent information on external sensory inputs. One possibility is that synchronous neural oscillations link multiple brain regions and contribute to the internal information representation. Neural oscillations orchestrate the temporal organization of neural firing and support communications between remote brain regions (Berens and Horner, 2017), which facilitates efficient encoding of information in distributed neuronal populations in millisecond ranges (Havenith et al., 2011).

fMRI and PET studies have attempted to delineate brain areas that are active during a specific stage of working memory processing (e.g., encoding), but it can be difficult due to the temporal resolution limitations of imaging modalities. However, electrophysiological studies of humans using electroencephalography (EEG) and magnetoencephalography (MEG) provide high temporal resolution, which helps to clarify the spatiotemporal dynamics of working memory processing. Many studies using EEG and MEG have demonstrated that neuronal oscillations closely relate to item encoding in working memory. For instance, one MEG study reported decreased gamma oscillatory power in the prefrontal area and increased beta oscillatory power in frontoparietal areas during encoding vibrotactile flutter in a working memory task (Von Lautz et al., 2017), suggesting a functional role of neural oscillations in the processing of abstract quantities. Greater alpha activity has also been found in the occipital and parietal regions *via* EEG recording during the encoding of visual items (e.g., arrow orientations) (Myers et al., 2014; Poliakov et al., 2014) or auditory items (e.g., verbal digits) in working memory tasks (Wilsch and Obleser, 2016; Wang et al., 2017), reflecting attentional control and active inhibition of irrelevant stimuli. These findings demonstrate that neural oscillations at different frequencies are important for item encoding in working memory as successful memory processing depends on sustained neural oscillations in the complex cortical network (Buzsáki and Draguhn, 2004; Palva et al., 2010).

In particular, neural oscillation at the theta frequency (4–8 Hz) is one of the best-studied rhythms and is prominent in the hippocampus and extrahippocampal regions (e.g., the prefrontal cortex and visual cortex). This oscillation has often been attributed to cognitive functions (e.g., attentional control) (Inanaga, 1998; Sauseng et al., 2007; Ishii et al., 2014) and is thought to be critical for successful memory functioning (Hanslmayr et al., 2019; Herweg et al., 2020). Several studies have shown that theta oscillations play an important role in working memory (Sauseng et al., 2007, 2010; Roux and Uhlhaas, 2014; Albouy et al., 2017; Esmaeili and Diamond, 2019) because low-frequency neural oscillations best correspond to working memory content (Lisman and Jensen, 2013; Roux and Uhlhaas, 2014). Moreover, it is well-known that the frontal theta amplitude is linearly correlated with working memory load (Raghavachari et al., 2001; Jensen and Tesche, 2002; Onton et al., 2005; Zakrzewska and Brzezicka, 2014). Theta oscillations have proved important during the encoding of different types of information in working memory. For instance, enhanced theta power was obtained across multiple cortical regions (e.g., prefrontal and temporal cortex) during the encoding of visuospatial or lexical items in working memory tasks (Sederberg et al., 2003; Sauseng et al., 2004; Jaiswal et al., 2010). These studies have shown consistently that theta oscillations are associated with encoding items from different domains in working memory.

Recently, analysis of functional connectivity (FC), defined as the temporal correlation between spatially distant neurophysiological events at the population level (Friston, 2011), has been increasingly applied to EEG data (Stam and Van Straaten, 2012). The human brain can be characterized as a “connectome,” which provides the anatomical substrate for information processing and reflects the internal state of brain activity (Sporns, 2011; Park and Friston, 2013). FC at frequency domains enabling communications between various neuronal populations through phase coherence (Fries, 2005) has been reported in working memory research using sensor- and source-level analysis (Babiloni et al., 2004; Zhang et al., 2016; Dai et al., 2017). For instance, studies have shown increased FC between the frontoparietal areas (electrode pairs: F4–P4, F3–P3) at beta and gamma frequency in a visual working memory task by sensor-level analysis (Babiloni et al., 2004).

However, little research has addressed the neural oscillation characteristics of multisensory item encoding in working memory. Our world is inherently multisensory and requires us to receive and process information from multiple sensory modalities simultaneously. To reach a realistic understanding of how working memory processes information temporarily from different sensory modalities, multisensory working memory research has become increasingly important (Quak et al., 2015). Several EEG studies have shown strong associations between neural oscillations and multisensory processing. For instance, enhanced gamma activity in the occipitoparietal regions has been found when participants performed an audiovisual object recognition task (Yuval-Greenberg and Deouell, 2007). In addition, increased theta activity in frontal-central areas has been observed during the processing of unisensory stimuli (auditory or visual stimuli) in contrast to bisensory stimuli (simultaneous auditory and visual stimuli) (Oliver et al., 2000).

Although evidence from EEG or MEG data suggests that neural oscillations in remote brain regions are an underlying mechanism of multisensory processing, the neural oscillation characteristics of multisensory item encoding in working memory remain largely unknown. The present study set out to investigate the sources of neural oscillations and their neural connectivity by using EEG to record neural oscillations when participants encoded complex audiovisual objects in a working memory task.

## Materials and Methods

### Participants

The participants in the experiment were 40 healthy adults selected from the local community (all right-handed; age range: 19–30 years; mean age: 26.34 years; 23 males and 16 females). Eligible participants did not use any medication that affected the central nervous system and had normal or corrected-to-normal visual acuity. All participants were informed about the experimental procedure and provided written informed consent before data collection, conforming to the tenets of the Declaration of Helsinki in all respects.

### Stimuli

To improve ecological validity, the stimulus materials were complex objects comprising natural pictures and sounds. The pictures were obtained from a standard set of outline drawings with 300 × 300 px resolution (Snodgrass and Vanderwart, 1980). The selected pictures contained a roughly equivalent number of objects from different semantic categories, including animals, tools, and instruments. The sounds were the vocalizations corresponding to the pictures (e.g., a vocalization of a dog barking corresponding to a picture of a dog). All sound files were taken from a website (http://www.fndsounds.com) and were modified using an audio-editing software (Adobe Audition 2018) with the following parameters: 16-bit resolution; 44.1-kHz samples; and a duration of 0.6 s with a 10-ms linear amplitude enveloping at sound onset and offset to prevent click effects. Three types of stimuli were used in the present study: single auditory (A), single visual (V), and congruent audiovisual object combinations (AV) (e.g., a picture of a dog matched with the barking of a dog).

### Task

**Figure 1:**
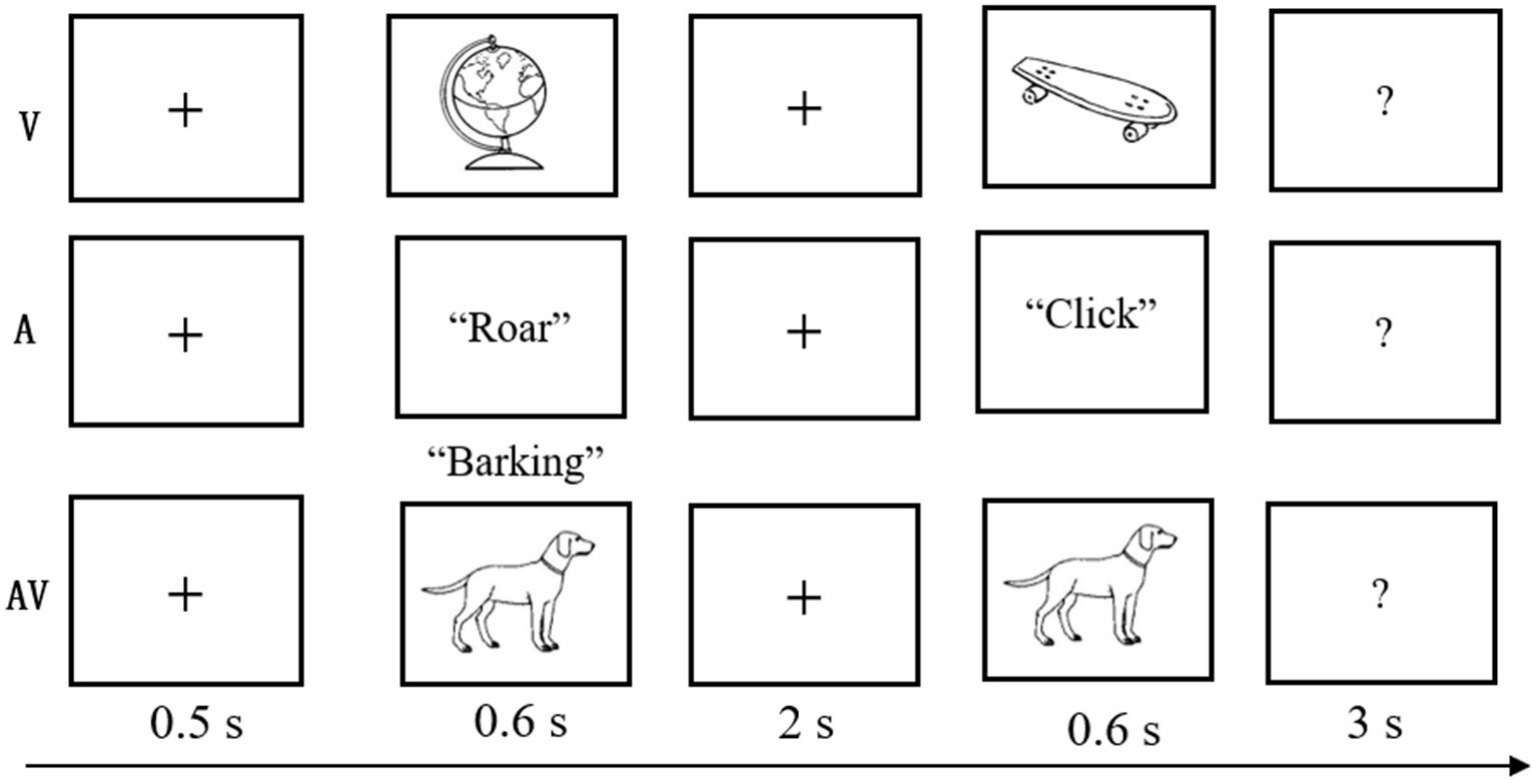
Schematic of the experimental task. A three-block design was used in the behavioral experiment. In each three-block trial, a fixation cross was shown for 0.5 s; a stimulus (visual-V, auditory-A, or congruent audiovisual-AV) was then presented for 0.6 s; a blank screen was shown for 2 s; and finally, the test stimulus appeared for 0.6 s with a 3-s time limit.

The experimental task was modified from a delayed match-to-sample paradigm (Sternberg, 1966), which involves presenting a series of memory items, a delay duration during which the information must be maintained, and probes evaluating the availability of the information after the delay. Details of this task are displayed in Figure 1. At the beginning of each trial, a fixation cross was shown for 0.5 s, followed by the encoding phase, during which time a stimulus (V, A, or AV) was presented for a duration of 0.6 s. After a blank screen of 2 s duration, a probe stimulus (single auditory or visual object) was displayed, which lasted for 0.6 s within the time window limit of 3 s. The visual objects were presented on a 17-in. computer monitor that subtended the visual angle by ~6.5° with a black background, and the auditory objects were presented binaurally at an intensity level of 75 dB through headphones.

Participants were asked to judge whether the probe stimulus was the same as the previously presented stimulus. The probe was novel in 50% of all trials. The intertrial interval ranged from 1,500 to 3,000 ms. The trials were presented in three blocks (A, V, and AV), and the sequence randomly varied between participants. Feedback was given at the end of each block, and breaks were encouraged between blocks to prevent fatigue. The experimental task was designed and controlled by E-Prime 2.0 (Psychology Software Tools Inc., Pittsburgh, PA; http://www.pstnet.com/eprime).

### Data Acquisition and Preprocessing

EEG data were recorded using the Neuroscan NuAmps system (sample rate: 1,000 Hz) with a 64-channel Quick-Cap (Compumedics Neuroscan Corp., USA). The electrodes were placed at the outer canthus (left and right) and the left eye (below and above) to record eye movements. Two additional reference electrodes were placed on the bilateral mastoids. All electrode impedance levels were kept below 5 kΩ on average.

EEG data were preprocessed and analyzed using EEGLab (Delorme and Makeig, 2004) (version 13.6, https://sccn.ucsd.edu/eeglab) and Brainstorm (Tadel et al., 2011) (https://neuroimage.usc.edu/brainstorm) running on a MATLAB platform (R2018a; MathWorks Inc., USA). The continuous EEG data were segmented from −0.2 to 0.6 s for a duration of 0.8 s around stimulus onset, as object encoding during the task (0.6 s duration) was the sole interest. All epochs were then zero padded to reduce edge effects of the relatively short signal. Offline band-pass filtering was performed using a fourth-order Butterworth filter with cut-off frequencies of 1 and 40 Hz and re-referenced to the average of the bilateral mastoid before further analysis.

Each epoch was then baseline corrected by subtracting the mean voltage before the sample stimulus. EEG epochs contaminated with strong muscle artifacts were manually rejected by visual inspection. The components containing blink and oculomotor or other artifacts were removed from the brain-driven EEG signals using an independent component analysis method. After preprocessing, an average number of 80.12 (SD = 10.28) trials per subject and block remained (A: M = 26.35, SD = 2.34; V: M = 25.36, SD = 3.41; AV: M = 24.17, SD = 4.39). A repeated measure analysis of variance (ANOVA) revealed no significant differences between the three blocks in the remaining number of trials (*F* = 1.26, *p* > 0.05).

### Data Analysis

#### Power Spectral Analysis

Power spectral analysis was performed on each epoch by applying a fast Fourier transform with a Welch window function and a window overlap ratio of 50%. The spectrum was calculated for windows of 0.6 s duration, and the frequency resolution was set at 1.67 Hz. The resultant power spectrum was averaged separately across trials for each condition. The spectral densities of all conditions were further acquired for measurement for global comparisons. Repeated ANOVAs were performed on global spectral density comparisons between all conditions. A *p*-value of < 0.05 was set for statistical significance using Bonferroni corrections for multiple comparisons.

#### Time-Frequency Analysis

Time-frequency decomposition was computed by convolving stimulus-locked single-trial data from all electrodes using complex Morlet wavelets. The wavelet cycles varied from 3 to 6 in logarithmically spaced steps to achieve comparable frequency precision at low and high frequencies. The time definition was from 0.2 to 0.6 s, and the frequency definition was linear from 1 to 40 Hz with steps of 1 Hz. Instantaneous power was estimated as the square of the complex convolution signal and averaged across trials. The resulting power was normalized with respect to its baseline (−0.2 to −0.01 s) and scaled with mean over baseline (dB). After spectral normalization, response transforms were averaged to produce the average time-frequency space. The differences in grand mean time-frequency representations between conditions (AV vs. V and AV vs. A) were computed across all subjects (*t*-test, *p* < 0.05; multiple comparisons by 1,000 randomizations with Monte-Carlo simulations).

#### Source Location Analysis

The exact low-resolution brain electromagnetic tomography (eLORETA) was used to estimate the intracortical source at frequency domains during the encoding phase of the working memory task. This method yields a distributed linear inverse solution that forms a current density map by minimizing the L2 norm of the difference between the observed data and the predicted forward solution.

The cross-spectral matrix was computed for each epoch and averaged within-subject and each condition. It was then submitted to the inverse solution algorithm, resulting in eLORETA maps at frequency domains. A different image of sources was computed by subtracting the A+V condition from the AV condition using voxel-by-voxel paired *t*-test. The nonparametric permutation test by randomization provides a single threshold statistical framework to account for the multiple comparisons problem [55], producing the maximum statistic of log-transformed *F* with a significant level for multiple tests at each voxel (non-parametric permutation test with 5,000 permutations, *p* < 0.05). The resulting different maps were then superimposed on a standard reference anatomical brain image for visualization.

#### Source-Based FC Analysis

The brain areas identified from the source locations by eLORETA were defined as regions of interest (ROIs) (10 mm radius sphere with MNI coordination). A lagged linear connectivity method implemented in eLORETA, which is based on intracortical lagged phase coherence through normalized Fourier transforms, was used to measure synchronous co-activation between any pair of ROIs at a given frequency (Pascual-Marqui, 2007). The method is considered to depict an accurate measurement of brain connectivity that is resistant to non-physiological artifacts and minimally affected by low spatial resolution in EEG data (Stam et al., 2007; Pascual-Marqui et al., 2011). The critical probability threshold value at a specific FC between two ROIs was determined by a paired sample *t*-test (AV vs. A and AV vs. V) with multiple comparison corrections across voxels (non-parametric permutation test with 5,000 permutations, *p* < 0.05).

#### Behavioral Performance Analysis

Repeated measurement of ANOVA was used to examine behavioral performance, including response time (RT) and accuracy rate (AR) across conditions using SPSS 20.0 (IBM, Corp., Armonk, New York). *Post-hoc* analyses using Bonferroni corrections accounted for multiple comparisons.

## Results

### Behavioral Performance

**Figure 2:**
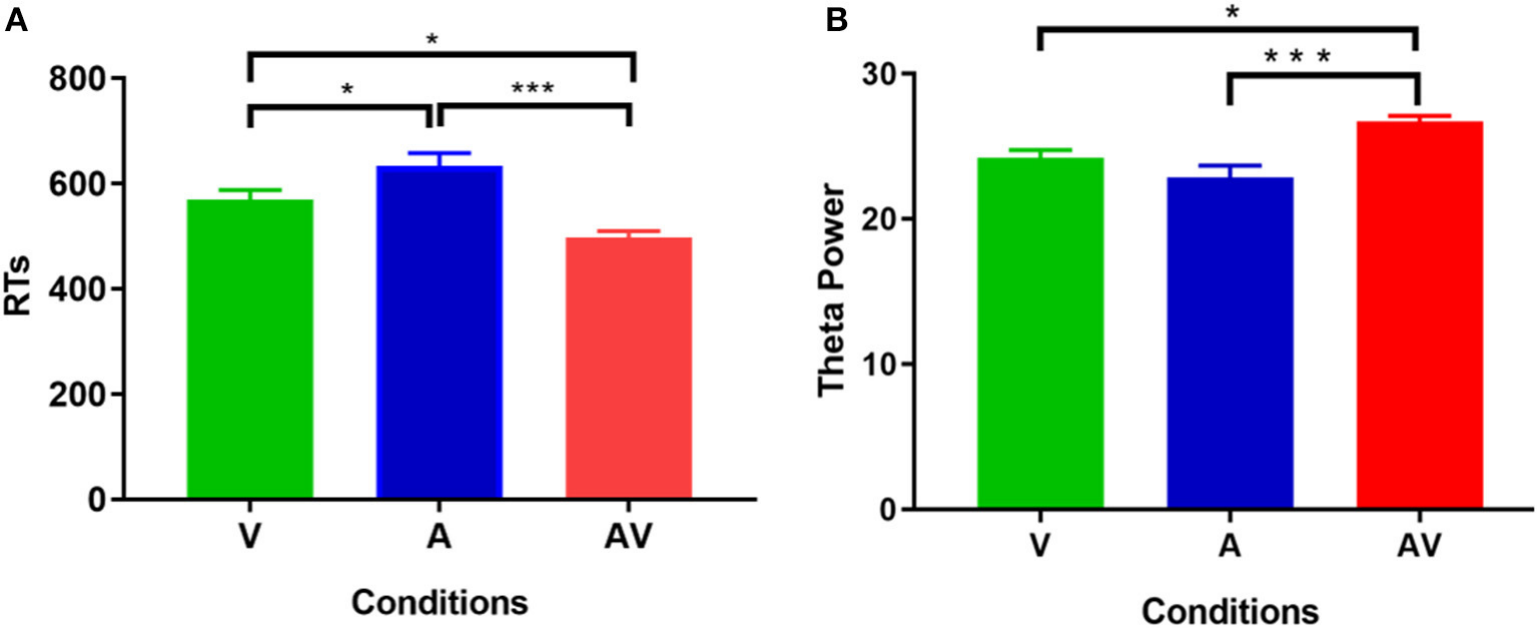
Statistical results of behavioral data. **(A)** Comparisons of RT for memory retrieval among the three conditions (V, A, and AV). **(B)** Comparisons of power spectral density in theta frequency among the three conditions (V, A, and AV) during the encoding phase of working memory processing (0–600 ms). The error bar presents the standard error of the mean. ^*^p < 0.05; ^***^p < 0.001.

The mean AR of memory retrieval was high (over 90%) in each condition; hence AR showed no significant difference among the three conditions (*F* = 1.87, *p* > 0.05). Nevertheless, mean RT of memory retrieval showed a significant difference across conditions (*F* = 21.19, *p* < 0.001). *Post-hoc* comparison showed that mean RT was faster in the audiovisual condition (M = 500.79 ms, SD = 76.88 ms) compared with the single auditory (M = 622.44 ms, SD = 143.67 ms, *t* = 5.11, *p* < 0. 001) and visual condition (M = 547.67 ms, SD = 95.46 ms, *t* = 2.39, *p* < 0. 05) (Figure 2A). In addition, Pearson correlation analysis revealed no significant association between theta power during the encoding period and RT at each condition (*p* > 0.102 for all). This result was similar to that of a previous study (Sghirripa et al., 2020), and it is possible that our task was not sufficiently difficult to capture the association.

### Power Spectrum

**Figure 3:**
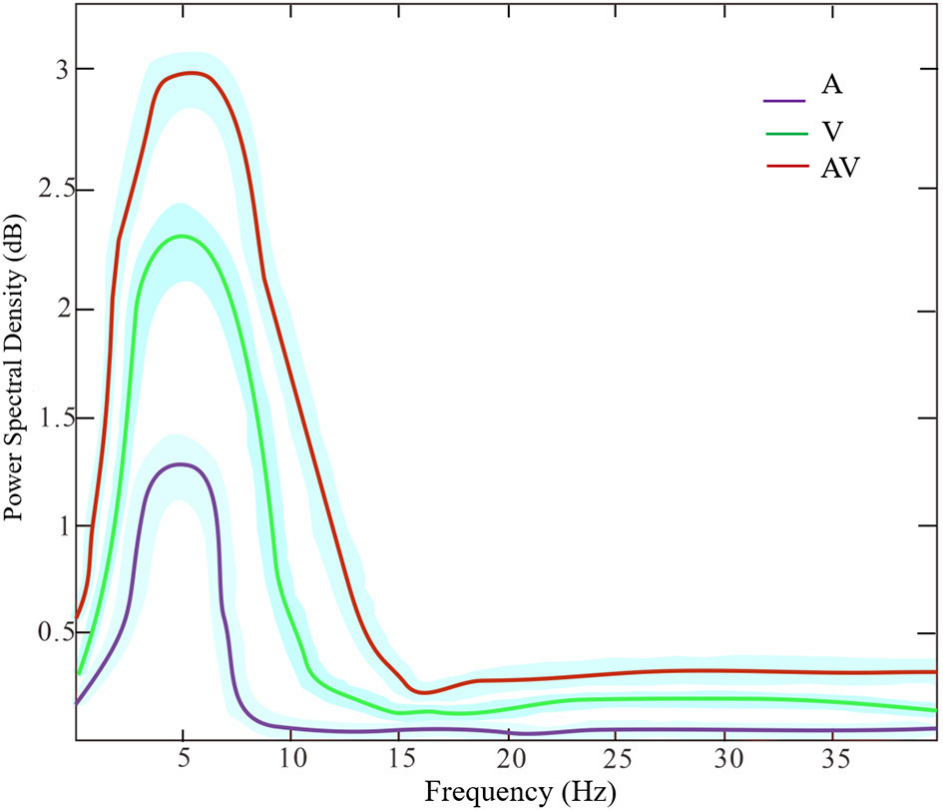
The power spectrum density of frequencies (peak at theta band around 5 Hz) across the three encoding conditions (A, V, and AV). The shaded area denotes the standard deviation.

Figure 3 shows the grand averages of EEG power spectrum across subjects under the three encoding conditions (A, V, and AV). As can be seen, there was a peak frequency of around 5 Hz (4–8 Hz) theta oscillation across the three conditions. The differences in spectral power density among the three conditions were significant (*F* = 11.87, *p* < 0.001). *Post-hoc* analysis showed that the global EEG power spectral density of theta oscillation in the AV condition (M = 26.68, SD = 2.49) was higher compared with the A condition (M = 22.84, SD = 5.00, *t* = 4.51, *p* < 0.001) or V condition (M = 24.21, SD = 3.18, *t* = 2.91, *p* < 0.05) (Figure 2B). Event-related potentials (ERPs) are considered as additional brain responses that reflect the engagement of working memory processing and the modulation of background oscillation power (Van Dijk et al., 2010). Thus, the effect of ERPs on theta oscillation power was additionally examined in this study (Supplementary Table 1), although the ERPs did not differ between the three conditions (Supplementary Figure 1).

### Time Frequency

**Figure 4:**
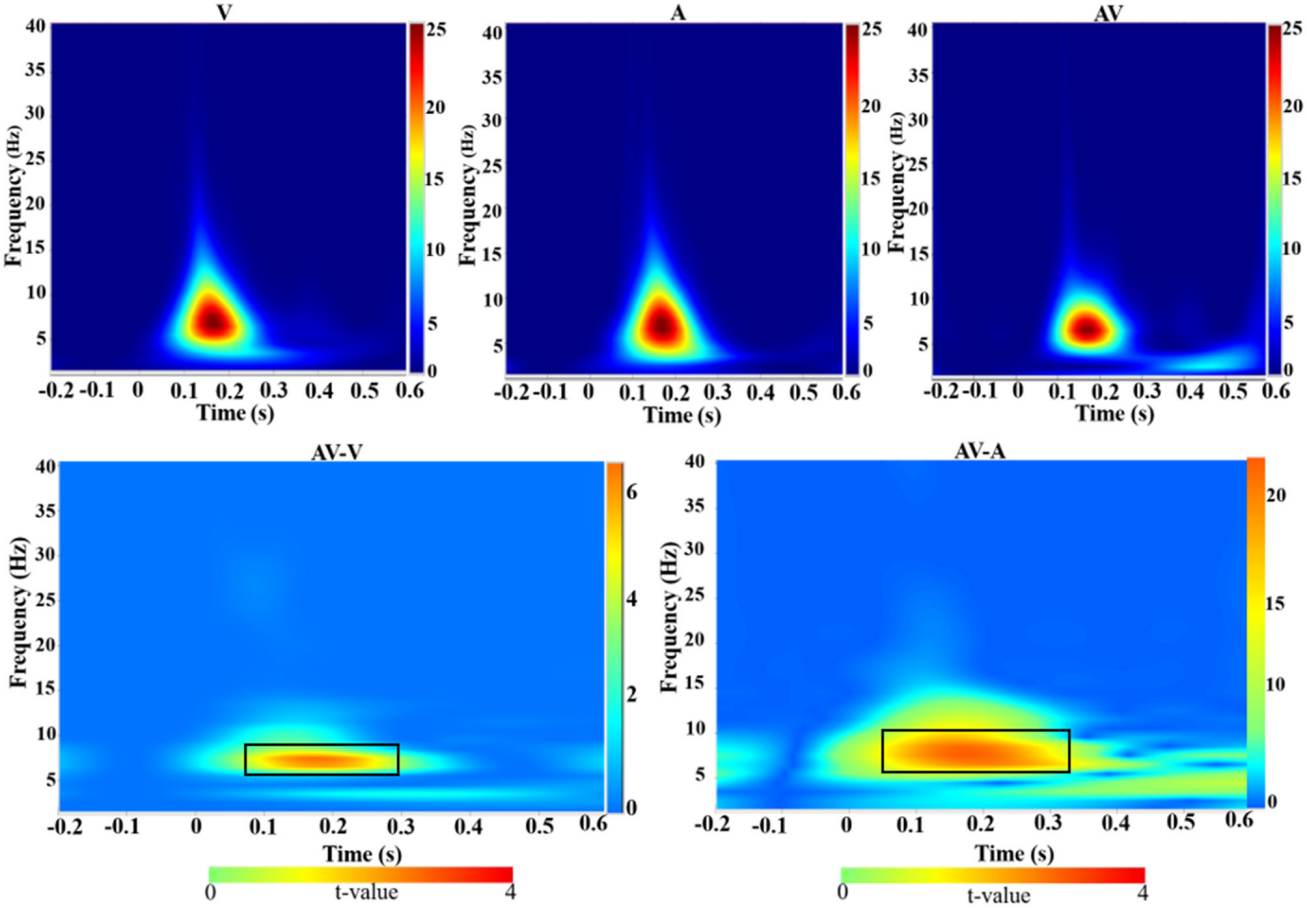
Group-averaged time-frequency spectra during the three conditions (V, A, and AV) and comparisons of the conditions (AV vs. V and AV vs. A). Time (in seconds) is denoted on the *x*-axis, with 0 s defined as the onset of the encoding stimulus. Frequency (in Hz) is shown on the *y*-axis. All signal power data are expressed in a logarithmic scale, with the color legend shown to the far right (unit: dB). Statistical values (*t* values) of time-frequency representation comparisons of the conditions are also shown with the color legend at the bottom. The top panel shows that theta activity increased during the encoding stage (110–270 ms) across the three conditions (V, A, and AV). The bottom panel, marked with a black box, shows the differences in theta activity between the AV and V conditions and between AV and A.

Time-frequency analysis revealed an increased theta activity (4–8 Hz) during the three encoding conditions (V, A, and AV) in the 110–276-ms time range (Figure 4, top panel). Theta activity also showed differences in enhancement between the conditions (AV vs. A: *t* = 4.51, *p* < 0.001; AV vs. V: *t* = 2.91, *p* < 0.05) (Figure 4, bottom panel).

### Source Locations at Frequency Domain

**Figure 5:**
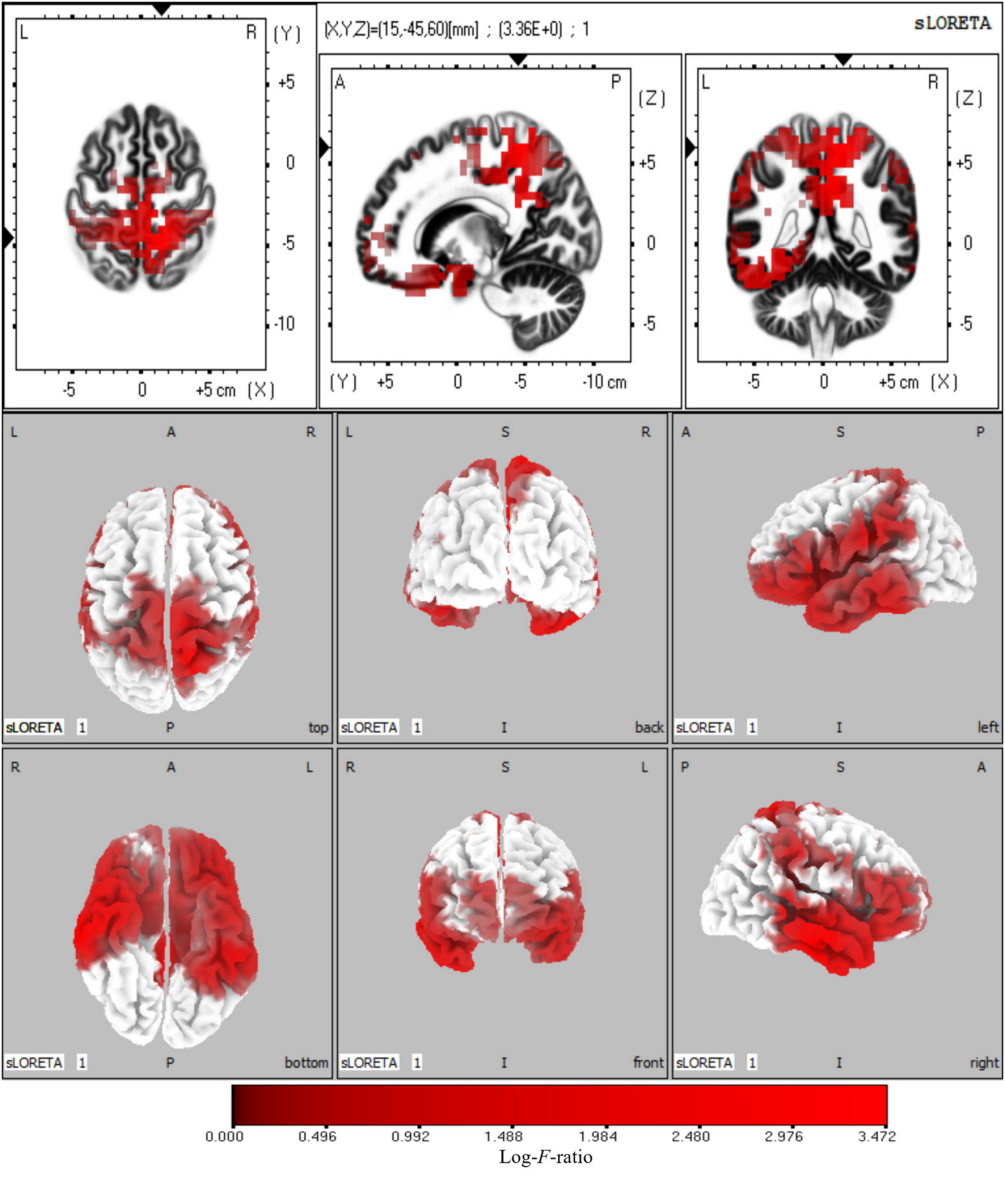
Statistical maps of source locations at theta frequency were projected onto a three-dimensional brain MRI template (top panel) and fiducial cortical surface (bottom panel). Non-parametric permutation test statistical analysis was performed to compare the current density distributions of AV with A + V. Colored areas represent the spatial extent of voxels with a significant difference in the current density. Log-*F*-ratio statistics were applied, and the color scale represents log-*F*-ratio values (threshold log-*F*-ratio = 3.36, *p* < 0.05). The MRI slices are located at MNI-space coordinates. In the averaged time windows of theta activity (110–260 ms), the maximum current density differences were found in the left superior frontal gyrus (Brodmann area 8, MNI: −30 20 50), right precuneus (Brodmann area 7, MNI: 5 −40 45), right inferior temporal gyrus (Brodmann area 21, MNI: 65 −15 −20), right cuneus (Brodmann area 18, MNI: 20 −95 20). The color scale represents the log-*F*-ratio values of current source density (threshold: log-*F*-ratio = 3.36, *p* < 0.05). A, anterior; P, posterior; S, superior; I, inferior; L, left; R, right.

The sources with significant power changes between AV and A conditions and between AV and V conditions at theta frequency were localized in widespread brain regions, including the left superior frontal gyrus, right inferior temporal gyrus, right precuneus, and right cuneus (log-*F-*ratio = 3.36, *p* < 0.05) (Figure 5).

### FC Based on Source Locations

**Figure 6:**
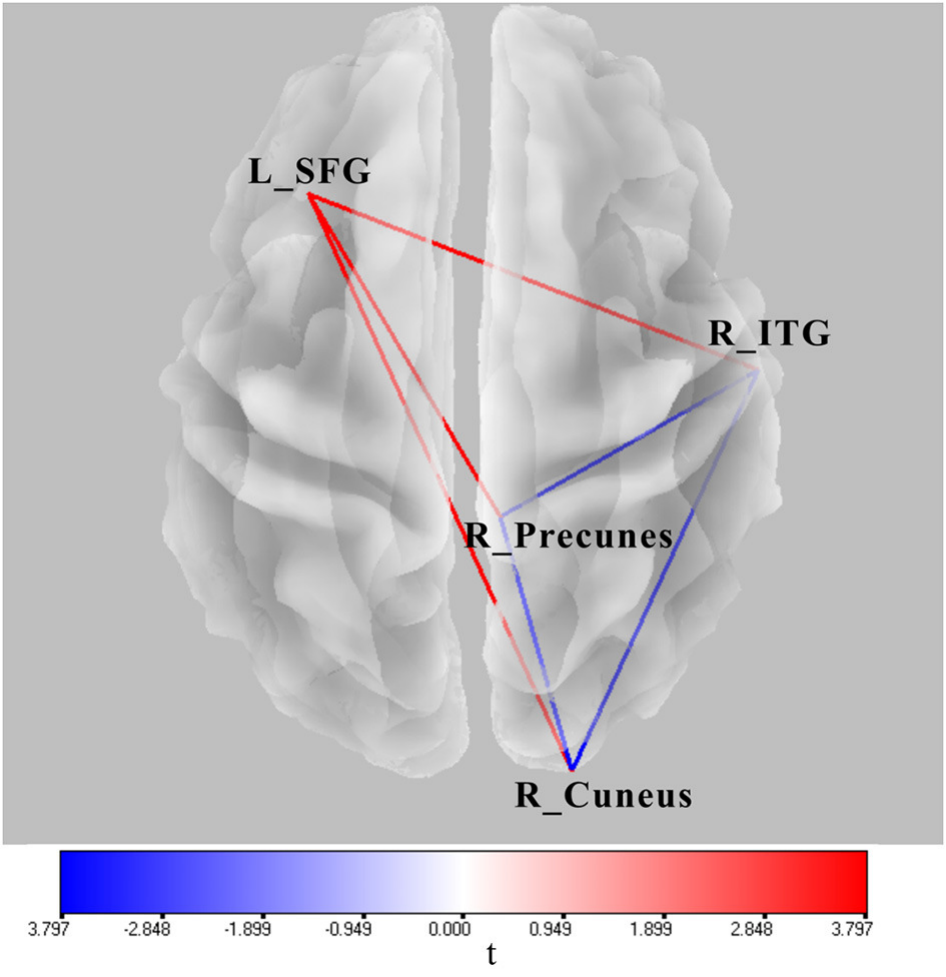
Wire diagram showing significant FC differences between AV and A conditions and between AV and A+V (|*t|*_min_ > 3.73, *p* < 0.05) (blue and red wires) based on physiological lagged connectivity measures by cortical eLORETA signals. These results correspond to theta oscillations (4–8 Hz). The red wire denotes increased connectivity; the blue wire denotes decreased connectivity. L, left; R, right; SFG, superior frontal gyrus; ITG, inferior temporal gyrus.

Significant lagged coherence changes in the ROIs were observed in the theta frequency. As compared with the superposition of A and V, significant increased lagged theta coherence was found in AV in several brain connections with the maximum, including the left superior frontal gyrus and right inferior temporal gyrus, left superior middle frontal gyrus and right precuneus, and left superior frontal gyrus and right cuneus (|*t|*_min_ > 3.73, *p* < 0.05) (Figure 6). Meanwhile, lagged theta coherence significantly decreased in AV compared with A+V across three brain areas: the right inferior temporal gyrus–right precuneus, right inferior temporal gyrus–right cuneus, and right precuneus–right cuneus (|*t|*_min_ > 3.73, *p* < 0.05) (Figure 6).

## Discussion

The current study used EEG recordings to investigate neural oscillations and functional brain connectivity during complex audiovisual object encoding in a working memory task. The behavioral results showed that audiovisual object encoding facilitated memory retrieval, compared with single auditory and visual object encoding, which is consistent with the previous findings (Goolkasian and Foos, 2005; Delogu et al., 2009). Moreover, theta oscillations were prominent when encoding audiovisual objects in working memory, and the sources of theta oscillation were widely distributed, including prefrontal (e.g., superior frontal gyrus), parietal (e.g., precuneus), temporal (e.g., inferior temporal gyrus), and occipital (e.g., cuneus) cortices. In addition, significant neural connectivity across cortical regions was observed in theta frequency domains. These results suggest that local theta oscillations and the corresponding neural connections play an important role during audiovisual encoding and may be responsible for the formation of working memory traces from multisensory items.

Neuronal oscillations are typical aspects of rhythmical brain activity (Fransen et al., 2015) that can combine neurons into assemblies to functionally support temporal information representation (Buzsáki and Draguhn, 2004) and play an important role in cognitive functions (Fell et al., 2003; Ward, 2003; Thut et al., 2006; Boucher et al., 2019; Prystauka and Lewis, 2019). Theta oscillation is an important frequency activity that is closely related to working memory (Tesche and Karhu, 2000; Jensen and Tesche, 2002; Sauseng et al., 2004; Onton et al., 2005; Raghavachari et al., 2006; Semprini et al., 2020; Yu et al., 2020). Studies of human EEG have shown that increased theta activity is associated with item encoding in working memory (Jensen and Tesche, 2002; Proskovec et al., 2019). Moreover, studies have provided causal evidence for the role of theta activity during working memory encoding by combining functional magnetic resonance imaging and rhythmic transcranial magnetic stimulation (Riddle et al., 2020). Theta oscillations likely serve as the “gluing mechanism” for human memories (Hyman et al., 2003) because they provide time windows for fast-acting long-term potentiation and depression (Hanslmayr et al., 2016). Evidence from multisensory memory research shows that theta oscillations enable episodic memories from different sensory inputs to be better integrated (Clouter et al., 2017). Our results suggest that theta oscillations are vital for audiovisual object encoding in working memory, which could then contribute to the unification of simultaneously presented auditory and visual objects into a single integrated sensory presentation, since neural oscillations are an essential mechanism to create a unified object feature from different sensory modalities (Senkowski et al., 2008). Additionally, encoding or integrating information across sensory features or modalities is more resource demanding than those of individual features or modalities (Humphreys, 2001; Wheeler and Treisman, 2002) because the encoding of audiovisual objects evokes stronger neural activity than single objects.

Working memory processing occurs across a distributed network of brain areas (Constantinidis and Klingberg, 2016), ranging from the sensory to the parietal and the prefrontal cortex (Christophel et al., 2017). Studies of humans and non-human primates have indicated that multiple cortical areas are associated with multisensory processing and are activated during the integration of audiovisual stimuli, including the temporal (e.g., inferior temporal gyrus), occipital (e.g., cuneus), and parietal (e.g., precuneus) cortices (Schneider et al., 2008; Erickson et al., 2014; Casado-Aranda et al., 2018). In addition, the prefrontal cortex is the neural basis of the central executive function of working memory, both controlling memory resources and supervising other memory components (D'esposito et al., 1995; Funahashi, 2017). Additionally, the prefrontal cortex participates in attentional control for audiovisual stimuli and allocates a limited capacity of memory resources to multisensory items (Anderson et al., 2010; Keller et al., 2017). As studies have indicated frontal theta activity is primarily involved in the allocation of attention toward target stimuli (Gomarus et al., 2006; Missonnier et al., 2006), neural oscillations across distributed brain regions may reflect large-scale communications in the cortical network and play an important role during audiovisual object encoding.

As neurons in the brain are highly interconnected (Sauseng and Klimesch, 2008), functional interactions among them could be a mechanism of information processing (Funahashi, 2006). Interactions between brain regions at the theta frequency have been reported in several studies of working memory, showing significant interregional theta synchronization involving the frontotemporal, frontoparietal, and fronto-occipital regions in auditory or visual item encoding (Sauseng et al., 2004; Kawasaki et al., 2010, 2014; Liebe et al., 2012; Muthukrishnan et al., 2020). Moreover, large-scale theta synchronization during working memory encoding is related to the co-activation of cortical networks and involves the critical coordination and integration of processes in the formation of memory traces (Wu et al., 2007; Sauseng et al., 2010). Consistent with these findings, we found increased connectivity of the prefrontal cortex with posterior cortices including temporal, parietal, and occipital regions.

Theta oscillations are relatively specific for control processes in working memory (Sauseng et al., 2010), as mentioned above, frontal theta connectivity reflects the central executive functions of working memory (Sauseng et al., 2006; Hanslmayr et al., 2007). It is considered, therefore, that prefrontal activity at theta frequencies is a critical hub that enables connection with other cortical regions when encoding multisensory items, because successful working memory functioning requires effective communication and the coordination of distributed brain networks (Cohen et al., 1997; Faw, 2003). In addition, the precuneus, inferior temporal gyrus, and cuneus are considered to be the critical nodes of the default mode network (DMN) (Spreng et al., 2008; Utevsky et al., 2014; Raichle, 2015), which routinely exhibits anticorrelations when performing attention-demanding cognitive tasks (Fox et al., 2005). It is not surprising that there was a decreased connectivity within the regions during audiovisual object encoding. According to the general hypothesis of DMN, individuals must disengage from self-related thoughts that may interfere with audiovisual object encoding, which is reflected by a decreased connectivity within the DMN network.

In conclusion, the results indicate that audiovisual object encoding in working memory is a complex cognitive process involving multiple brain areas and is reflected correspondingly by interregional theta connectivity. The results also indicate that local theta oscillations and their long-range neural connectivity form the underlying mechanism for audiovisual object encoding in working memory.

## Data Availability Statement

The original contributions presented in the study are included in the article/Supplementary Material, further inquiries can be directed to the corresponding author.

## Ethics Statement

The studies involving human participants were reviewed and approved by the Ethics Committee of Xin Yang College. The participants provided written informed consent to participate in this study.

## Author Contributions

YX conceived and planned the research. YX and YL wrote the manuscript. YX, HD, XX, and PF performed the analyses. YX, YL, WZ, and PF discussed the results and contributed to the final manuscript. All authors contributed to the article and approved the submitted version.

## Conflict of Interest

The authors declare that the research was conducted in the absence of any commercial or financial relationships that could be construed as a potential conflict of interest.

## Supplementary Material

Supplementary Figure 1 Comparisons of event related potential (EPR) components between the three conditions (A, V, and AV). The amplitude of N1 (100-160ms) and P2 (170-230ms) components did not differ between the conditions (p >0.136 for all).

Supplementary Table 1 Regression parameters form models predicting the amplitudes of N1 and P2 with theta power.
