# Supplementary figures and images for: Theta Oscillations and Source Connectivity During Complex Audiovisual Object Encoding in Working Memory
Source: Front Hum Neurosci. 2021 Mar 8;15:614950. doi: 10.3389/fnhum.2021.614950 (PMC7982740; doi:10.3389/fnhum.2021.614950)

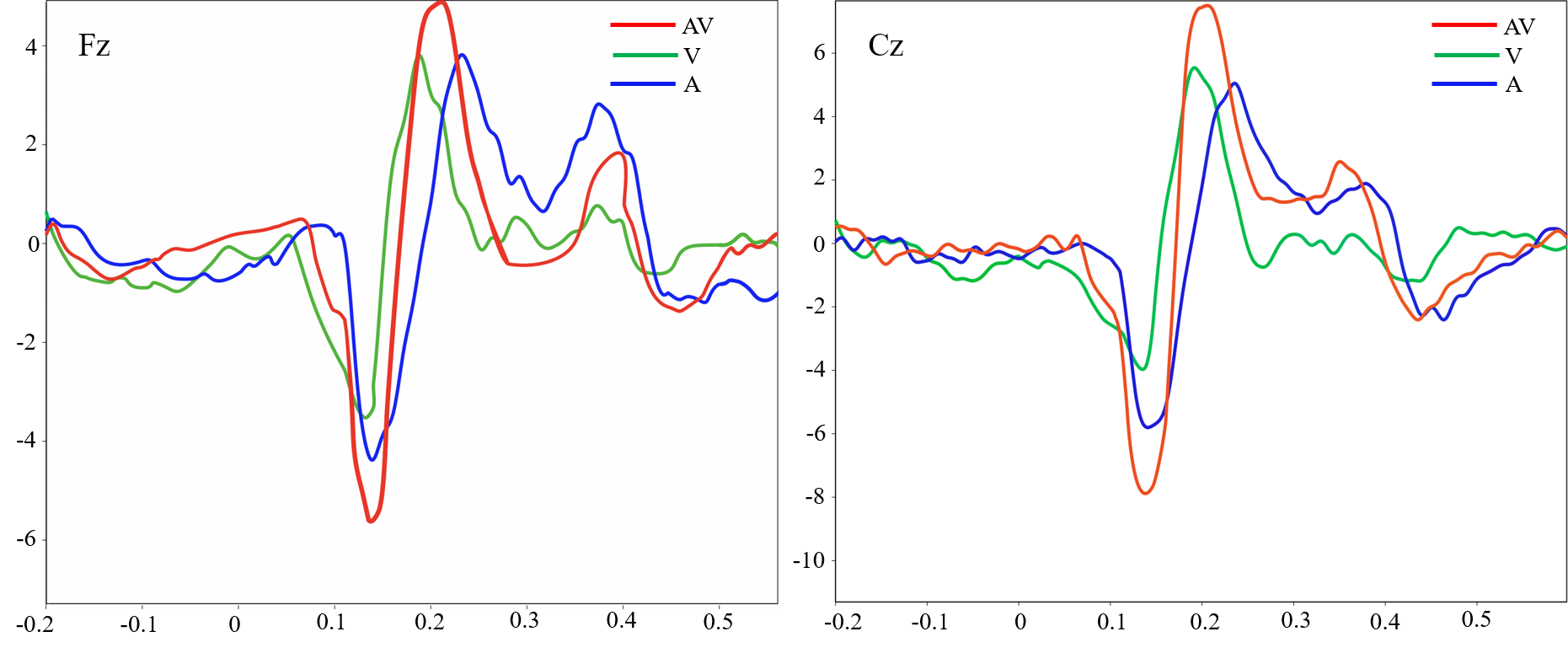

Supplement: Supplementary Figure 1 — Comparisons of event related potential (EPR) components between the three conditions (A, V, and AV). The amplitude of N1 (100-160ms) and P2 (170-230ms) components did not differ between the conditions (p >0.136 for all). [file Image_1.TIF]
