# Supplementary material for: Theta Oscillations and Source Connectivity During Complex Audiovisual Object Encoding in Working Memory
Source: Front Hum Neurosci. 2021 Mar 8;15:614950. doi: 10.3389/fnhum.2021.614950 (PMC7982740; doi:10.3389/fnhum.2021.614950)
Supplement: Supplementary Table 1 — Regression parameters form models predicting the amplitudes of N1 and P2 with theta power. [file Table_1.docx]

**Supplementary Table 1** Regression parameters form models predicting the amplitudes of N1 and P2 with theta power

| Component | Sensor | SE | t | *p* | 95% CI | |
| --- | --- | --- | --- | --- | --- | --- |
|  |  |  |  |  | Lower upper | Upper |
| N1 | Fz | 0.239 | 0.523 | 0.604 | -0.364 | 0.614 |
|  | Cz | 1.546 | -0.084 | 0.934 | -3.288 | 3.028 |
| P2 | Fz | 0.901 | 0.569 | 0.573 | -1.327 | 2.354 |
|  | Cz | 1.256 | -0.386 | 0.702 | -3.051 | 2.080 |

Note: Regression analyses were performed to assess whether theta power contributed and accounted for the N1 and P2 components. For each regression, theta power served as the predictor variable, with N1 and P2 alternately serving as the dependent variables. For each model, the overall grand average values across three conditions (V, A ,and AV) were used for the components and the theta power score.
